# Supplementary material for: Functional Genomics Identified Novel Genes Involved in Growth at Low Temperatures in Listeria monocytogenes
Source: Microbiol Spectr. 2022 Jun 23;10(4):e00710-22. doi: 10.1128/spectrum.00710-22 (PMC9431668; doi:10.1128/spectrum.00710-22)
Supplement: Supplemental file 1 — Supplemental material. Download spectrum.00710-22-s0002.pdf, PDF file, 0.7 MB [file spectrum.00710-22-s0002.pdf]

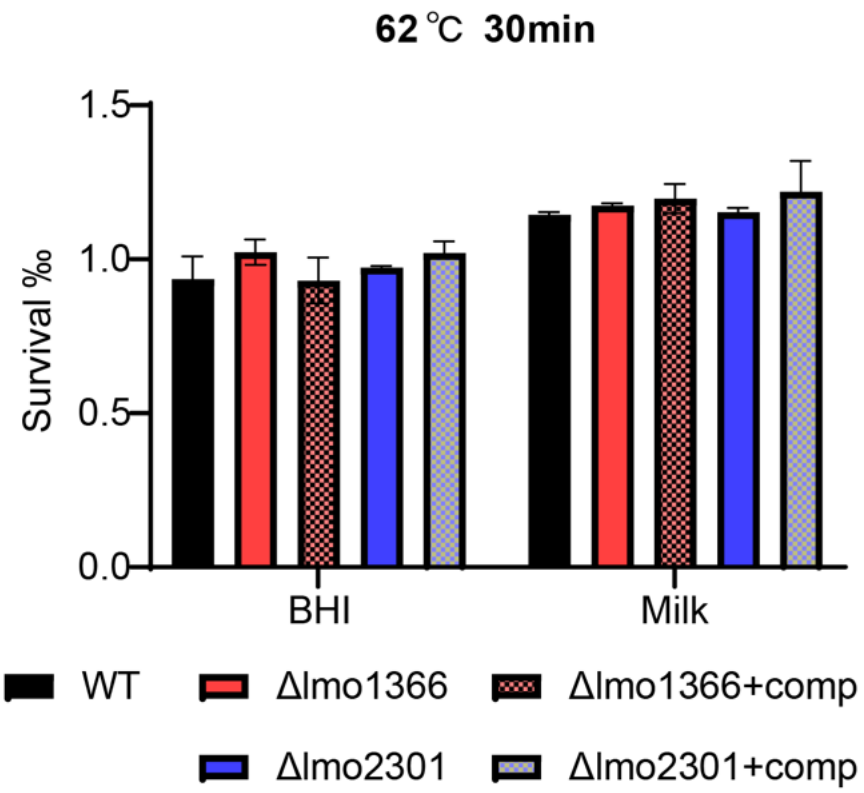

Fig. S1. Survival rate (‰) of strains at 62 °C after 30 mins in BHI or milk.

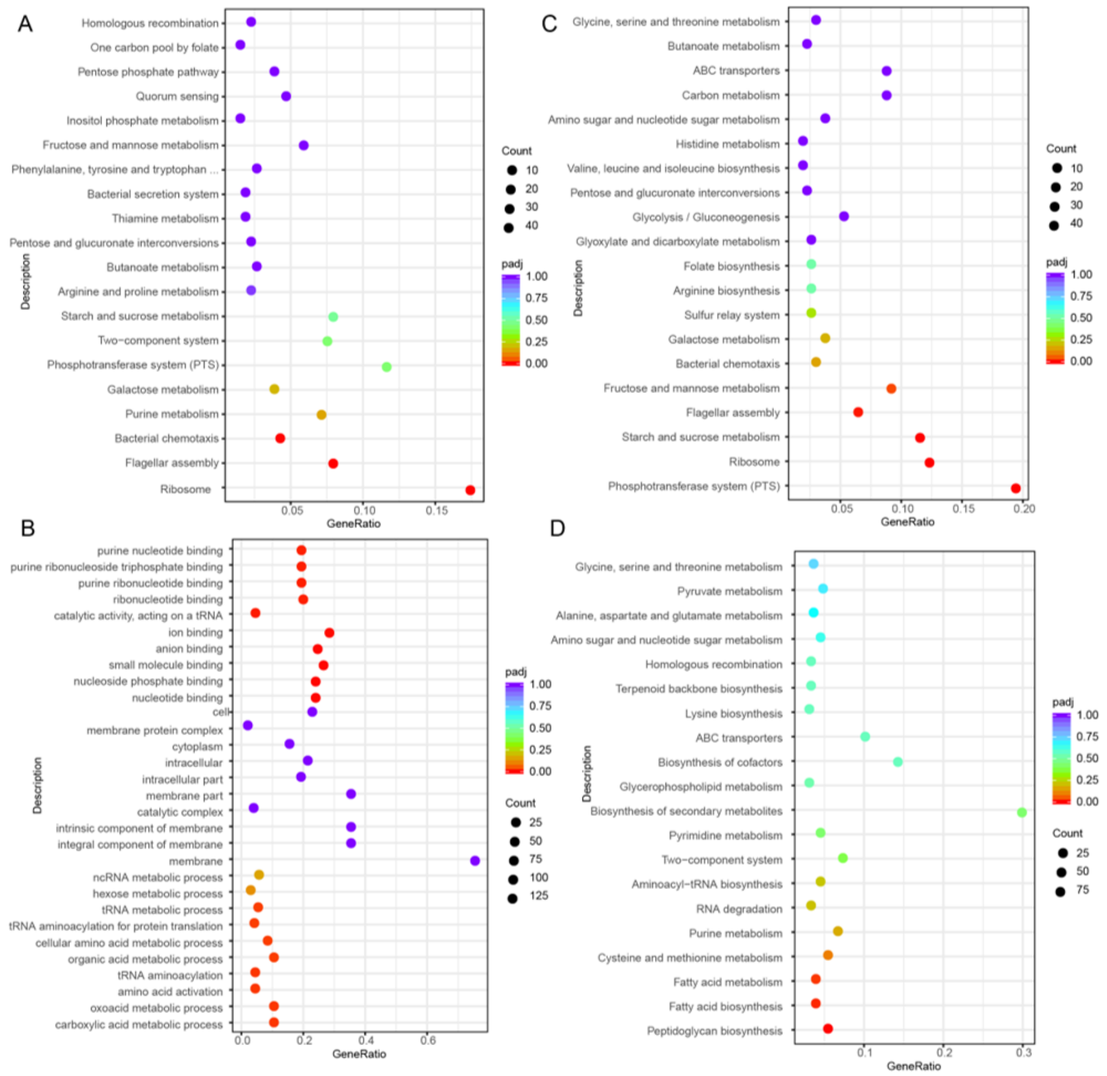

Fig. S2. KEGG or GO results of differential expressed genes of Δ*lmo1366* and Δ *lmo2301* versus wild-type strains at 16 ℃. **(A)** KEGG results of genes down-regulated of Δ*lmo1366* at 16 ℃. **(B)** GO results of genes up-regulated of Δ*lmo1366* at 16 ℃. **(C)** KEGG results of genes down-regulated of Δ*lmo2301* at 16 ℃. **(D)** KEGG results of genes up-regulated of Δ*lmo2301* at 16 ℃.

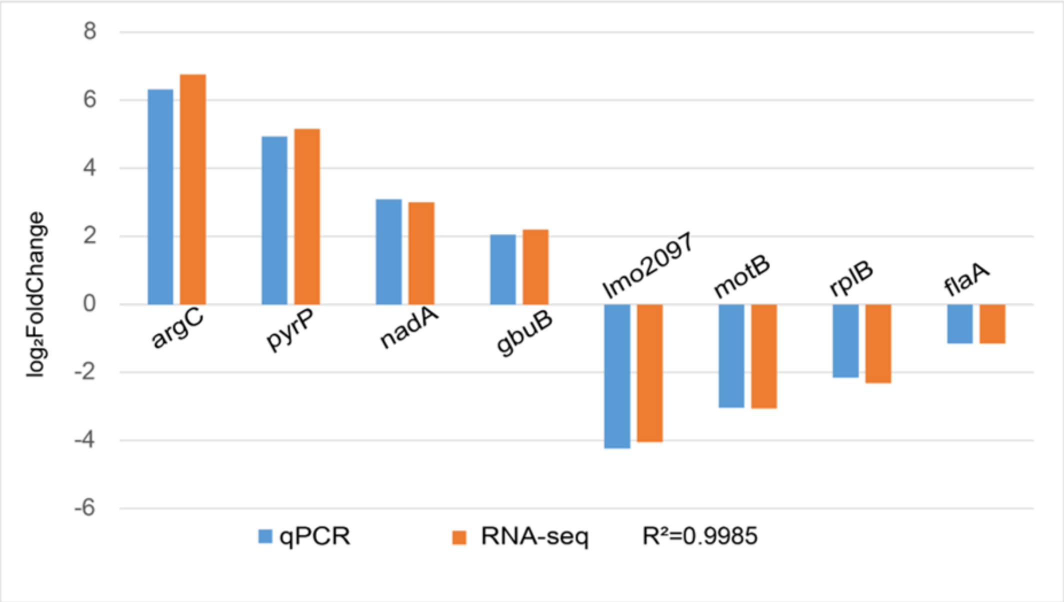

Fig. S3. qPCR verification of selected RNA-seq data. A housekeeping control gene drm normalized the gene expression ratios obtained from qPCR and RNA-Seq. Each gene was performed with three biological replicates. The y axis represented the value of Log2FC.

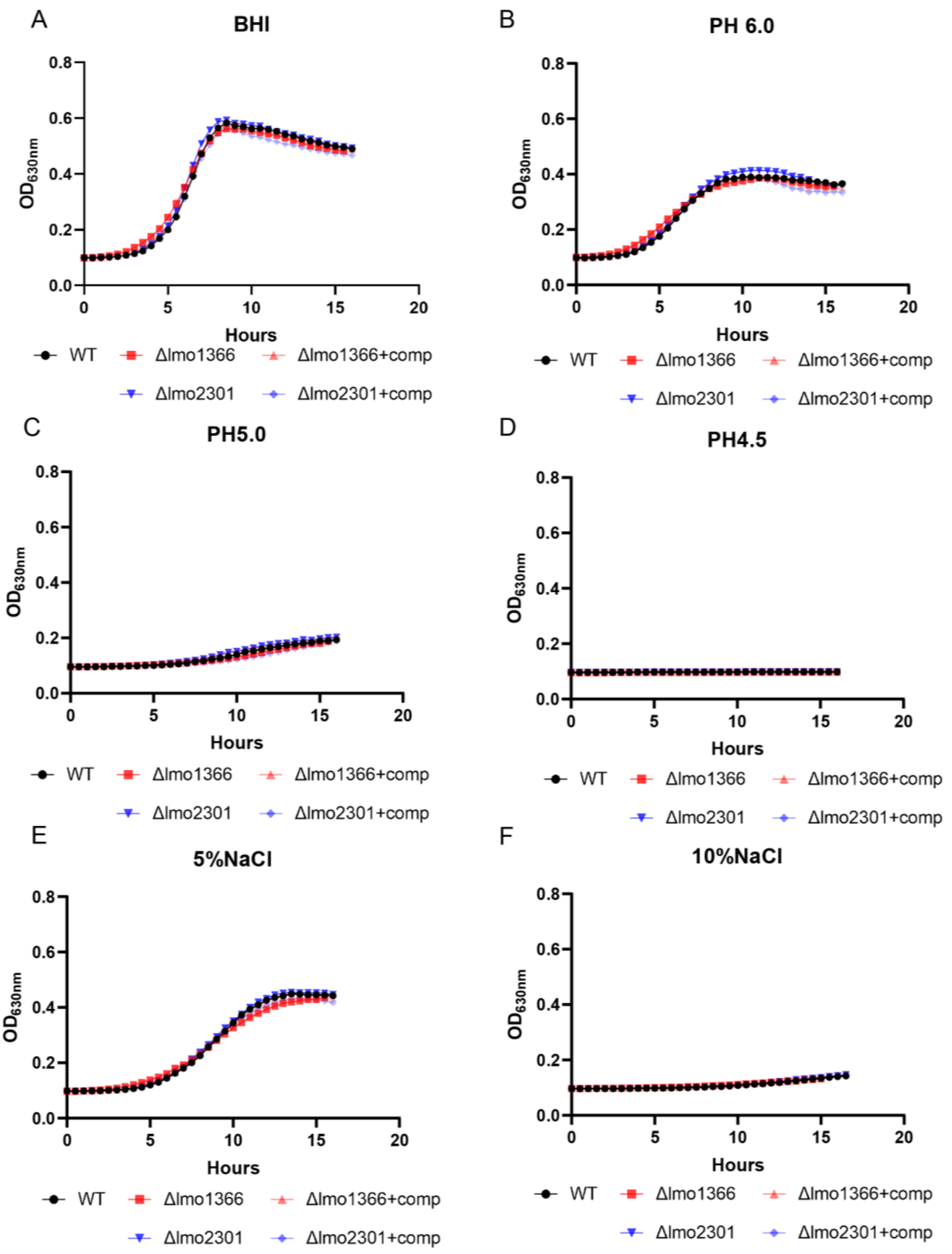

Fig. S4 Growth curves of the WT (●), Δlmo1366 (▲), Δlmo1366+comp (■), Δlmo2301(▼), Δlmo2301+comp (◆) in BHI at 37°C with PH (7.0, 6.0, 5.0, 4.5) and with NaCl (5%, 10%). Three biological replicates for each experiment.
